# Juxtaposition of the atrial appendages: A nidus for thrombus in atriopulmonary Fontan?

**DOI:** 10.21542/gcsp.2016.19

**Published:** 2016-06-30

**Authors:** Veronica Spadotto, Inga Voges, Philip J. Kilner, Magdi H. Yacoub, Sabine Ernst, Siew Yen Ho, Sonya V. Babu-Narayan

**Affiliations:** 1 NIHR Cardiovascular Biomedical Research Unit, Royal Brompton and Harefield NHS Foundation Trust, National Heart and Lung Institute, Imperial College, London, UK; 2 Department of Cardiac, Thoracic and Vascular Sciences, University of Padua, Padua, Italy; 3 Justus-Liebig-University of Giessen, Pediatric Heart Center, Giessen, Germany; 4 Department of Cardiac Morphology, Royal Brompton and Harefield NHS Foundation Trust

## Abstract

Juxtaposition of atrial appendages is a rare cardiac congenital anomaly, usually associated with other cardiac malformations. Until now, it has not been linked to any significant clinical implications. We report cardiovascular magnetic resonance (CMR) findings of two adult patients who underwent atriopulmonary Fontan operation in the setting of left juxtaposition of the atrial appendages. The patients were in sinus rhythm at the time of the CMR study. Both patients had episodes of sustained atrial tachyarrhythmia requiring electrical cardioversion and were anticoagulated with warfarin with target INR 2-3. CMR images showed a thrombus located in the enlarged and juxtaposed right appendage in both patients. Blood flow frequently appears slow or sluggish in the dilated right atrium following atriopulmonary Fontan surgery. In addition, cine CMR suggested that blood flow reaches very low velocities in the massively dilated juxtaposed right atrial appendage cul-de-sac, thus potentially creating a substrate for clot formation. These findings propose that juxtaposed atrial appendages in atriopulmonary Fontan is an additional risk factor for clot formation, specifically in the dilated right atrial appendage on the left side juxtaposed with the left atrial appendage and that prophylactic anticoagulation is highly justified in these patients.

## Introduction

Juxtaposition of the atrial appendages whereby both atrial appendages are located on the same side of the great arteries was first described in 1893^1^ and is a rare congenital anomaly occurring in 0.81% of congenital heart disease patients^2^. It is often associated with other complex cardiac malformations, especially cyanotic congenital heart diseases^3^. Left-sided juxtaposition appears to be much more frequent than right juxtaposition^4,5^. Transposition of the great arteries can be found in 50–92%, and tricuspid in 30–40% of patients according to different reports^3,4,6^. To date there have not been any significant clinical consequences.

## Clinical cases

We report findings in two adult patients with atriopulmonary Fontan for univentricular physiology both with juxtaposition of the atrial appendages. In neither case was the left-sided right atrial appendage directly anastomosed to the pulmonary artery.

**Figure 1.**
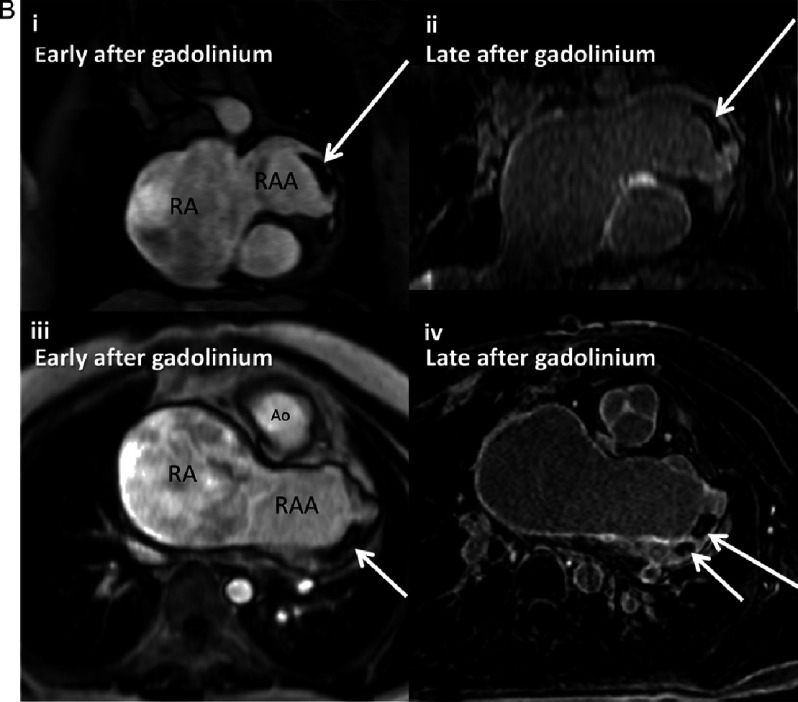
(A) Patient 1 imaging findings. (i) Chest radiography in posteroanterior view, showing bulging (white arrows) of left heart contour below the left pulmonary artery, as a result of left juxtaposition of the atrial appendages. Situs solitus is inferred from the normal bronchial anatomy and cardiomegaly is noted. (ii) Corresponding coronal image from 3D balanced steady state free precession (3D bSSFP). Grossly dilated right atrium and enlarged, left juxtaposed right atrial appendage (white arrows) characterised by pectinate muscles are noted. The darker spot amongst the pectinate muscles is thrombus - see Figure 1B. (iii) Right atrial dilatation with sluggish blood flow on still frame from cine CMR, axial view, also demonstrating the underlying tricuspid atresia. (iv) 3D bSSFP sagittal image showing dilated right atrial appendage (RAA) in the left hemithorax. Underlying transposition of great arteries can be noted with anterior aorta from the RV. CMR, cardiovascular magnetic resonance; RA, right atrium; RAA, right atrial appendage; Ao, aorta; PA, pulmonary artery; LPA, left pulmonary artery; RV, right ventricle; LV, left ventricle; LA, left atrium. **(B) Contrast-enhanced CMR findings documenting thrombus in patient 1.** Early after gadolinium injection, coronal (i) and axial views (iii) show dilated right atrium and appendage and a filling defect (dark region, white arrow) at the left tip of the right atrial appendage which is typical of thrombus. Corresponding coronal (ii) and axial (iv) image planes confirm low signal (darker) in the same region again consistent with thrombus within the right atrial appendage, (white arrows). CMR, cardiovascular magnetic resonance; RA, right atrium; RAA, right atrial appendage; Ao, aorta.

Patient 1 was a 27-year-old female, whose original cardiac condition was situs solitus, left juxtaposition of the atrial appendages, univentricular atrioventricular connection with absent right atrioventricular connection (tricuspid atresia), small anterior rudimentary right ventricle, unrestrictive ventricular septal defect, transposed great arteries lying anterior and to the left of pulmonary artery. She underwent pulmonary banding at the age of 6 months and had atriopulmonary Fontan procedure (right atrium to pulmonary artery anastomosis posterior to and to the right of the aorta) at the age of 6 years. Cardiovascular magnetic resonance (CMR) features of the underlying anatomy and chest x-ray features of left juxtaposed atrial appendages were seen (Figure 1A)^7^.

**Figure 2.**
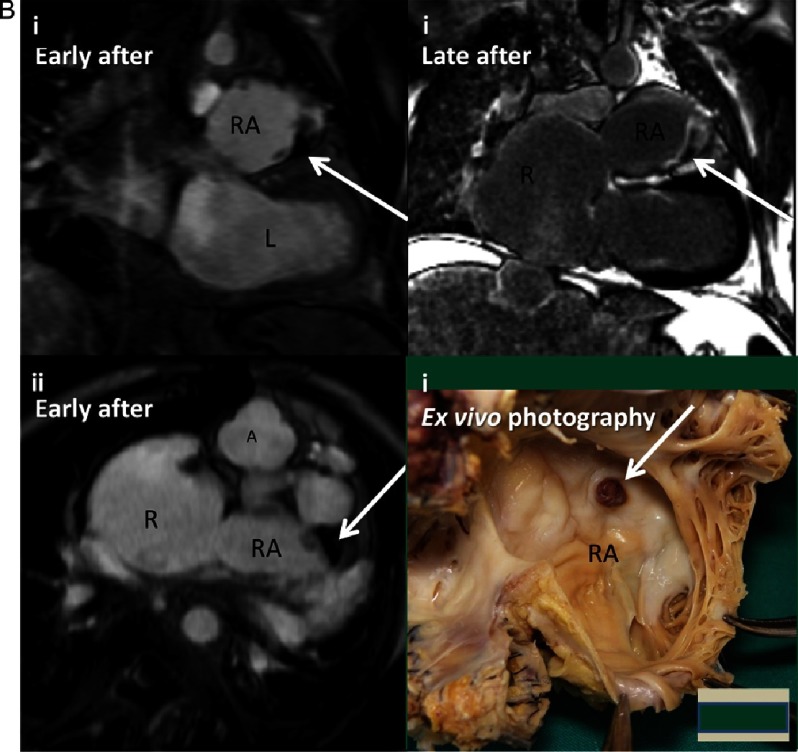
(A) Patient 2 imaging findings. (i) Chest radiography in posteroanterior view. As for patient 1, left juxtaposition of the atrial appendages produces a bulging left heart contour (white arrows). Situs solitus, cardiomegaly and scoliosis are also noted. (ii) Corresponding coronal image from 3D bSSFP for direct comparison with 2Ai. The left juxtaposed right atrial appendage (white arrows) appears almost of the same size as the already dilated right atrium. (iii) Right atrial dilatation with sluggish blood flow on still frame from cine CMR, axial view, similar to the findings for patient 1. The arrow points to the surgical patch to close off the right atrial to right ventricle connection. (iv) 3D bSSFP sagittal image showing dilated right atrial appendage (RAA) towards the left side. Underlying transposition of great arteries can be noted with anterior aorta from the RV. CMR, cardiovascular magnetic resonance; RA, right atrium; RAA, right atrial appendage; Ao, aorta; PA, pulmonary artery; LPA, left pulmonary artery; RV, right ventricle; LV, left ventricle; LA, left atrium. (B) **Contrast-enhanced CMR and ex-vivo findings documenting intra-auricular thrombus in patient 2.** Early after gadolinium injection, coronal (i) and axial (iii) images show, as for patient 1, a dark thrombotic region at the tip along the outer wall of the right atrial appendage (white arrows). The corresponding late gadolinium coronal image is again consistent with intra-auricular thrombosis (white arrow). Ex-vivo evaluation after explant for heart transplant (iv) confirmed the presence of a thrombus (white arrow). CMR, cardiovascular magnetic resonance; RA, right atrium; RAA, right atrial appendage; Ao, aorta; LV, left ventricle (Courtesy of Professor Siew Yen Ho).

Patient 2 was a 46-year-old male, born with situs solitus and left juxtaposition of atrial appendages, left ventricle, a large, non-restrictive ventricular septal defect, normally related great arteries with pulmonary stenosis. He underwent atriopulmonary Fontan procedure at the age of 9 years, with division of the main pulmonary artery and homograft valved conduits incorporated in the superior vena cava, inferior vena cava, and at the right atrium to distal main pulmonary artery connection. Subsequently, and prior to the adult CMR study shown, inferior vena cava anastomosis and right atrium-pulmonary artery connection stents were implanted at 45 years of age. CMR features of the underlying anatomy and chest x-ray features of left juxtaposed atrial appendages were seen (Figure 2A)^7^.

At the time of the CMR scan (1.5 Tesla, Siemens, Erlangen) both patients were in NYHA class I–II and were electively orally anticoagulated for primary prevention of thromboembolic events. Both patients had experienced episodes of sustained atrial tachyarrhythmia that required electrical cardioversion within hours of onset.

In patient 1, CMR showed a severely enlarged right atrium (88 × 70 mm) with a small thrombus within the tip of the dilated RA appendage distributed along the outer wall and neighbouring pectinate muscles and demonstrated on cine, early and late gadolinium study (Figure 1B).

In patient 2, CMR showed severe enlargement of the right atrium (90x76 mm) and the right atrial appendage, A thrombus within the tip of the right atrial appendage along the outer wall (Figure 2B) was documented for the first time during 15 years of follow up with periodic CMR surveillance shortly after he discontinued chronic oral anticoagulation treatment for 25 days due to haemoptysis.

## Discussion

We report on two patients with atriopulmonary Fontan and juxtaposition of the atrial appendages. Besides confirming the diagnosis of juxtaposition of the atrial appendages in detail, CMR demonstrated thrombus located in the dilated right atrial appendage juxtaposed with the left atrial appendage in both patients. Both patients had a history of atrial tachycardia. It is striking that both patients presented with intracardiac thrombus unusually located within the right atrial appendage.

There is swirling, forward and backward flow in the dilated right atrium post atriopulmonary Fontan operation (see Movie) with especially sluggish blood flow into and within the juxtaposed right atrial appendage on the left hand side. Anatomically, the latter appears to act as a static pouch or cul-de-sac into which slow flow is directed and in which blood recirculates, thus providing a nidus for thrombus formation. Future 4D flow phase-contrast imaging in patients with juxtaposition of the atrial appendages and previous atriopulmonary Fontan could also be of interest the present cine CMR images already show these flow patterns.

A recently published study showed that Fontan patients anticoagulated with warfarin but with poor control have increased risk for thrombosis formation compared to those with well-controlled warfarin therapy^8^. We note that one of our two patients with INR almost always in the desired therapeutic range (INR 2-3) developed new thrombus in the juxtaposed right atrial appendage after only 3 weeks of discontinued therapy.

We suggest that the clinical indication for prophylactic anticoagulation with good therapeutic control is particularly strong in the rare setting of Fontan patients with juxtaposed atrial appendages.

## Disclosures

The authors have no conflicts of interest.
